# An Australian convective wind gust climatology using Bayesian hierarchical modelling

**DOI:** 10.1007/s11069-023-06078-8

**Published:** 2023-08-08

**Authors:** Alessio C. Spassiani, Matthew S. Mason, Vincent Y. S. Cheng

**Affiliations:** 1 School of Civil Engineering, The University of Queensland, St Lucia, QLD 4072 Australia; 2 Climate Research Division, Science and Technology Branch, Environment and Climate Change Canada, Toronto, ON M3H 5T4 Canada

**Keywords:** Wind gusts, Climatology, Convective storms, Bayesian hierarchical modelling, Australia, Reanalysis

## Abstract

To quantify the hazard or risks associated with severe convective wind gusts, it is necessary to have a reliable and spatially complete climatology of these events. The coupling of observational and global reanalysis (ERA-Interim) data over the period 2005–2015 is used here to facilitate the development of a spatially complete convective wind gust climatology for Australia. This is done through the development of Bayesian Hierarchical models that use both weather station-based wind gust observations and seasonally averaged severe weather indices (SWI), calculated using reanalysis data, to estimate seasonal gust frequencies across the country while correcting for observational biases specifically, the sparse observational network to record events. Different SWI combinations were found to explain event counts for different seasons. For example, combinations of Lifted Index and low level wind shear were found to generate the best results for autumn and winter. While for spring and summer, the composite Microburst Index and the combination of most unstable CAPE and 0–1 km wind shear were found to be most successful. Results from these models showed a minimum in event counts during the winter months, with events that do occur mainly doing so along the southwest coast of Western Australia or along the coasts of Tasmania and Victoria. Summer is shown to have the largest event counts across the country, with the largest number of gusts occurring in northern Western Australia extending east into the Northern Territory with another maximum over northeast New South Wales. Similar trends were found with an extended application of the models to the period 1979–2015 when utilizing only reanalysis data as input. This implementation of the models highlights the versatility of the Bayesian hierarchical modelling approach and its ability, when trained, to be used in the absence of observations.

## Introduction

Weather and climate extremes have a major impact on society and infrastructure. Approximately 94% of all disaster losses are attributable to some form of severe weather events, with convective wind gusts (i.e. thunderstorms, downburst, and tornadoes) accounting for 41% of these events (Munich 2016). Within Australia, convective wind gusts are responsible for 50% of all wind-related damage to buildings (Blong 2005). Such impacts highlight a need to improve our understanding of this hazard so the risks they pose to the built environment and to society can be accurately quantified and mitigated.

A spatially complete climatology, based on robust and reliable data, is essential for improving the understanding of convective wind gust risk. Spassiani and Mason (2021) attempt to develop such a climatology for Australia based solely on Automatic Weather Station (AWS) data. To do this, they used the machine learning technique of Self-Organizing Maps to identify convective gusts from historic wind records. Their climatology showed that the frequency of convective gusts exceeding 90kmh^−1^ peaked at a count of ~ 3 per year over the northern coast of Western Australia (WA). Smaller maxima over southern Northern Territory (NT) (~ 1 per year) and in northeast New South Wales (NSW) (~ 1.5 per year) were also observed. In addition, they show that the majority of events occur during the summer months.

Brown and Dowdy (2021a) analysed severe wind gusts measured at 35 weather stations across Australia and used lightning data to classify which gusts were convective. They also analysed severe convective wind gust reported in the Australian Bureau of Meteorology (BoM) Severe Thunderstorm Archive (STA) (http://www.bom.gov.au/australia/stormarchive/), near these 35 stations. Their measured dataset shows convective wind gust counts of 0.6–1 per year to occur at most coastal stations in the southeast of the country, with this frequency extending north to the NSW-Queensland (QLD) border. They also show a few stations through central Australia with elevated event counts (~ 0.6 per year), generally showing similar trends to those found by Spassiani and Mason (2021), albeit with smaller absolute event counts. When comparing these observations with STA reports though, Brown and Dowdy (2021a) point to clear observational biases that lead to peaks in the highly populated cities around the country. This observation is similar to that found for convective storm reports in other parts of the world (Etkin and Leduc 1994; King 1997; Doswell 2001; Anderson et al. 2007; Allen et al. 2011). Such sampling bias has historically been accounted for with statistical models that correct observations in areas with low population density (Twisdale 1982; Tescon et al. 1983; King 1997; Ray et al. 2003). More recently, Bayesian statistical models have been utilized to correct for observational biases within meteorological datasets (Anderson et al. 2007; Cheng et al. 2013; Elsner and Widen 2014; Elsner et al. 2016; Potvin 2019; 2022) due to their ability to incorporate uncertainty (Arhonditsis et al. 2007; 2008a,b) and address complex natural systems (Clark 2005;Cheng et al. 2020). However, Cheng et al. (2013) show there remains high levels of uncertainty when applying such corrections to large areas with unreliable or non-existent observations (such as exist over much of Australia).

Considering this, it is often difficult to develop an accurate climatology based on wind gust observations alone. Additionally, requirements for high fidelity data collected over an extended period (standard practice, as outlined by WMO (2017), is 30 years) and the fact that observational networks are generally sparse when compared with the small spatial scale of convective wind events (Brooks et al. 2003a) also add to this difficulty. To overcome these limitations, rather than studying the event observations themselves, other studies have sought to analyse the environments that produce severe weather (e.g. Brooks et al. 2003b; Grunwald and Brooks 2011). This approach assumes there is a strong relationship between the occurrence of severe convective winds and the broad scale atmospheric conditions that lead to their development. Such relationships can be determined for areas where severe weather observations are reliable and then extended to areas with less reliable observations. Reanalysis data (e.g. ECMWF ERA-Interim, ERA5) have typically been used as a reliable measure of atmospheric conditions in these studies and given the global nature of these data thunderstorm climatology information has been developed for much of the world (e.g. Brooks et al. 2003a; Allen et al. 2011; Allen and Karoly 2014; Taszarek et al. 2019).

For Australia, this type of analysis has been undertaken for environments conducive to general severe convective thunderstorm activity (Kuleshov et al. 2002; Dowdy and Kuleshov 2014; Allen and Karoly 2014; Bedka et al. 2018), or more specifically for individual convective thunderstorm hazards, such as hail (Soderholm, et al. 2017; Dowdy et al. 2020) or convective wind gusts (Brown and Dowdy 2021a). For their investigation of convective winds, Brown and Dowdy (2021b) used the logistical regression model developed in Brown and Dowdy (2021a) to explain the environments associated with severe convective wind gust in Australia. They also suggest that this diagnostic is suitable for analysing long-term variability of severe convective wind environments and show an annual increase in the number of severe convective wind gusts, using the global climate model ensemble median, of 17%, 13%, 2%, and 2% in northern Australia, the rangelands, eastern Australia, and southern Australia, respectively.

Despite the success of these studies, it remains difficult to find atmospheric indices or combinations of these indices that capture all the necessary conditions for development of severe thunderstorms or their sub-event hazards, such as convective wind gusts. There are two further limitations with this type of climatological analysis; (1) it is only useful for analysing the relative storm hazard between areas as it does not fully account for the fact that meeting a threshold does not guarantee the development of a storm, and (2) it assumes the same covariate threshold is applicable across a range of climatological regions.

As an alternative to the development of a wind gust climatology reliant solely on either direct observation (e.g. Spassiani and Mason 2021) or analysis of severe weather environments (e.g. Brown and Dowdy 2021a, b), Bayesian hierarchical modelling offers an approach where both sets of information can be used (Cheng et al. 2015; 2016). Cheng et al. (2015) developed a Bayesian hierarchical modelling framework to develop a tornado climatology utilizing reanalysis-derived severe weather indices and tornado occurrence observations. Within this framework, tornado occurrence rates were corrected for observational biases in regions of low population using a Bayesian modelling approach to link observed tornado counts to severe weather environments and the probability of detection. Cheng et al. (2016) then used their Bayesian hierarchical model framework to develop annual and seasonal climatologies of tornado occurrence across North America. They also developed an approach to account for the role of regional variability in the occurrence of tornadoes through local model error terms that account for factors unable to be captured by the SWI, such as, local processes that can influence the convective initiation.

Given the success Bayesian hierarchical models have had improving tornado climatology estimates for North America (which has many similar observation density issues that face Australia), it seems reasonable to extend this approach to the estimation of convective gusts in Australia. As such, the objective of this paper is to develop a spatially complete climatology of severe convective wind gusts for Australia using Bayesian hierarchical modelling, reanalysis data, and weather station observations. This is done using a modified version of the Bayesian hierarchal model described in Cheng et al. (2016) with relationships between Severe Weather Indices (SWI) calculated using ERA-Interim reanalysis data and AWS wind gust observations used to predict seasonal and annual severe convective wind gust occurrence across Australia. Research methods, model formulation and data sources are discussed in Sect. 2. Model output results for different model combinations (i.e. the use of different SWI combinations) are presented and discussed in Sect. 3 and suggestions for the model combination that provides the most realistic climatology is provided. The work is then summarized in Sect. 4.

## Data and methods

### Observation data

More than 600 Automatic Weather Stations (AWS) are currently operated by the Australian Bureau of Meteorology (BoM). AWS record data at 1-min intervals, and the wind data recorded includes 1-min mean wind speed, peak 3-s gust within each 1-min period and mean wind direction. Data record lengths vary at each station, but many are short and given the land area of Australia, this network presents a spatially incomplete picture of the wind gust climate. Two main types of bias exist. The first is the influence of station density on report distribution. For example, rural areas with few or no stations will have an artificial minimum in reports. The second is an artificial increase in wind gust reports across the dataset over time due to a growing number of AWS stations in the observational network.

**Fig. 1:**
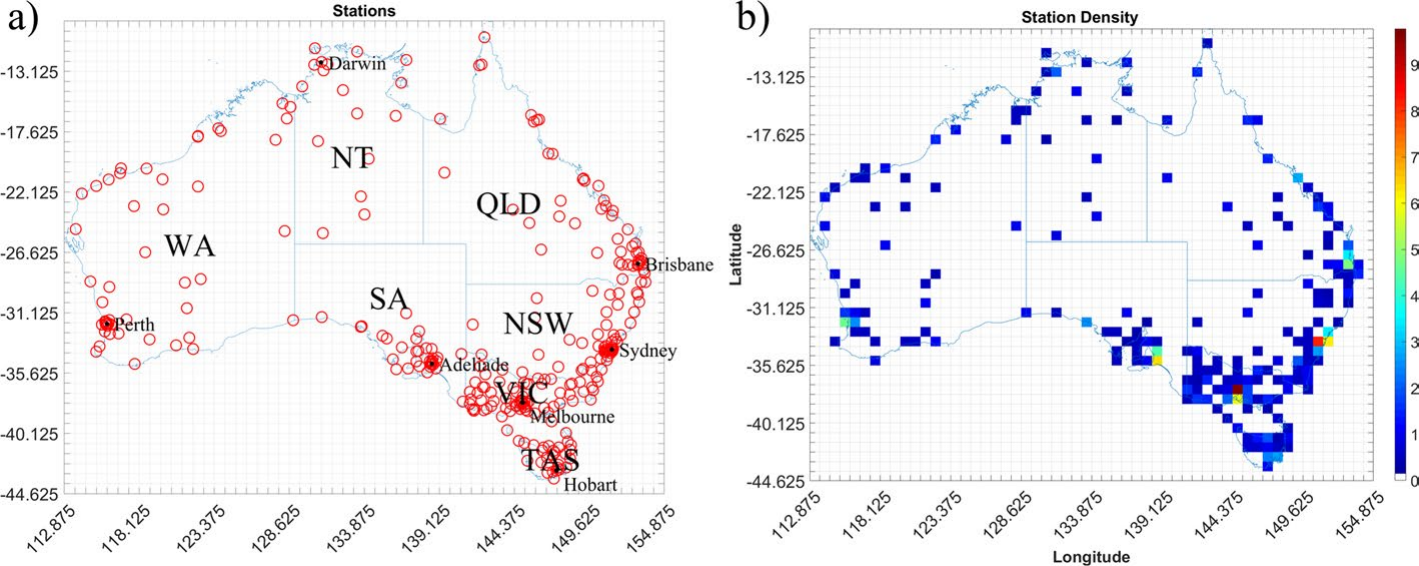
**a** Automatic Weather Station locations, **b** mean yearly AWS density for each ERA-Interim grid cell over Australia from 2005 to 2015

For this work, only stations with at least 5 years of 1-min data during the period 2005—2015 are considered. This leaves 306 stations for analysis, with the location of these shown in Fig. 1a and the time average density of weather stations within each analysis grid cell shown in Fig. 1b. From these stations, wind gusts exceeding 70kmh^−1^ (19.4 ms^−1^) were identified and the maximum of these observed within any given 6-h time block (00–06Z, 06-12Z, 12-18Z, and 18-00Z) were marked as gust events. While the choice of 6-h time blocks is somewhat arbitrary, this period is chosen to ensure that multiple events occurring on the same day, but resulting from different storm events, can be identified and included in the dataset. Gust wind speeds are then corrected to account for topographic influences following the approach specified in AS/NZS1170.2 (Standards Australia 2012) and described in detail in Spassiani and Mason (2021). After applying the topographic correction only wind gust exceeding 90kmh^−1^ (25 ms^−1^) are kept to be consistent with the BoM’s definition of a severe wind gust. Data are then quality controlled to removed fictitious events following the de-spiking method of Højstrup (1993), which was shown by Suomi et al. (2017) to effectively remove unrealistically high wind speeds from anemometer records. Any extended periods of erroneous or missing data not picked up by the de-spiking procedure were manually identified and removed before summating available data periods.

**Fig. 2:**
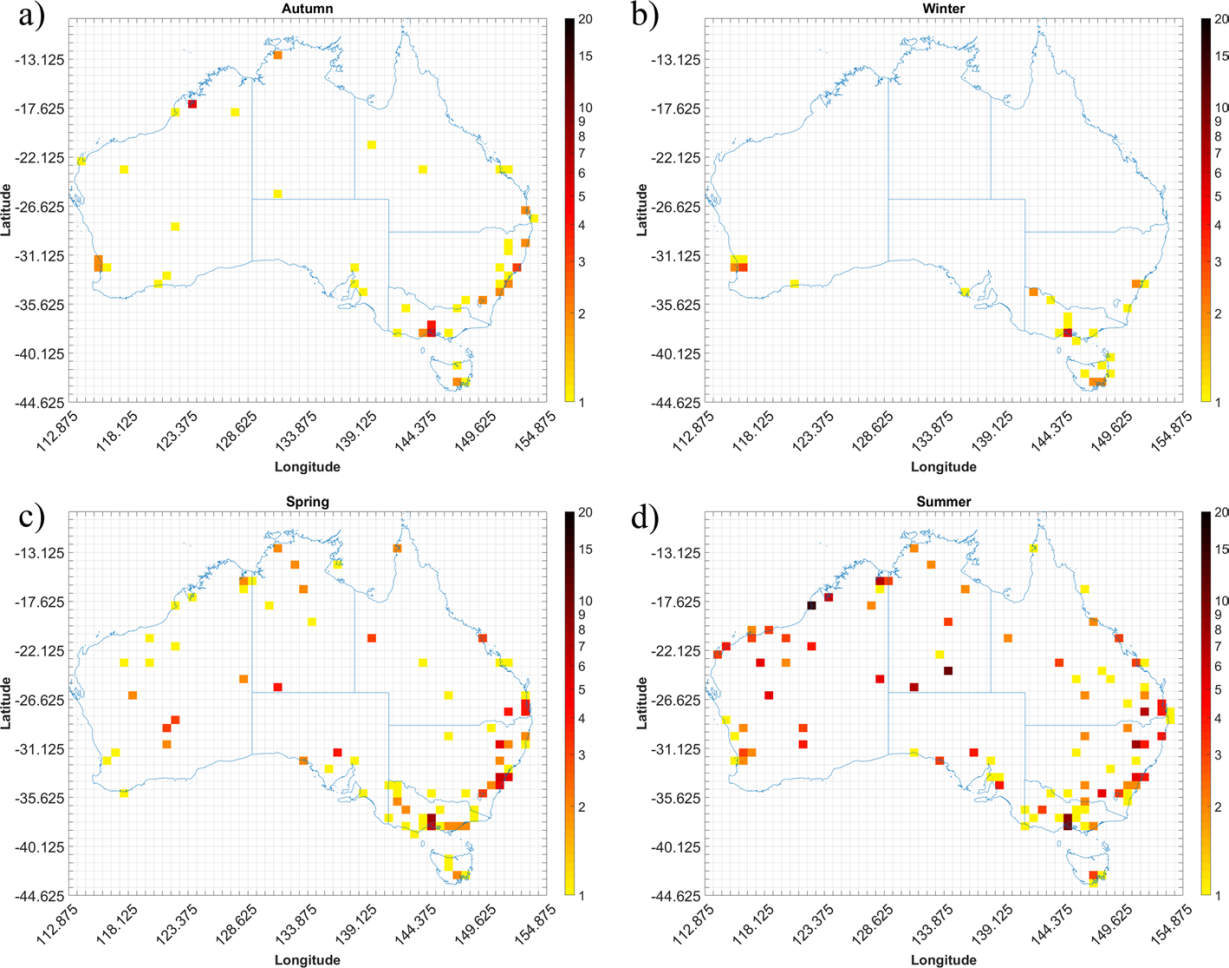
Number of days with at least one convective event observed within a grid cell between 2005 and 2015 for southern hemisphere **a** autumn, **b** winter, **c** spring, **d** summer

Following this procedure, 902 gust events were identified. Using the Self Organizing Maps (SOM) classification scheme outlined in Spassiani and Mason (2021), each event was then classified as either convective or non-convective in origin. In brief, this approach uses a 4X4 SOM that considers the wind speed, temperature, and pressure of each event to map it to a node in the SOM that closest represents the given event. The event is classified as convective if the probability of an event being convective for that given node is greater than 50%. This model was shown to identify convective wind gust events above 90kmh^−1^ with a mean absolute error of 0.11 events per year for the overall yearly event counts. Using these classified gust events, the number of convective gust events recorded within each of the analysis grid cells (i.e. ERA-Interim 0.75° grid) over Australia for the period 2005–2015 were determined. Where more than one station within a grid cell measured a given gust event, these “redundant” events were removed and the count of observed convective gusts events for the four Southern Hemisphere seasons; summer (December – February), spring (March–ay), winter (June–August), autumn (September–November) were aggregated, Fig. 2.

Spassiani and Mason (2021) note that there is some sensitivity in the classification of events as either convective or non-convective due to the choice of SOM and the convective event probability threshold. When examining another SOM, with similar performance skills, that considered the wind speed, wind direction, temperature, pressure, and precipitation, Spassiani and Mason (2021) note only small differences, specifically in WA where there was a decrease of ~ 0.5 convective events per year and a small increase in some station in TAS of ~ 0.25 convective events per year. This model was found to have a mean absolute error of 0.13 events per year for the overall yearly event counts.

The density bias resulting from larger numbers of AWS around population centres and low numbers in rural areas is evident through the station density plot (Fig. 1b) and the event density plots (Fig. 2) with the majority of events shown to occur near major cities. We find most events occur during the summer across Australia, with spring showing a fair number of events as well, especially along the eastern coast from Brisbane down to Melbourne. Less events occur during autumn with a minimum of events occurring in winter, with these confined to the southern half of Australia. More detailed analysis of these observations can be found in Spassiani and Mason (2021).

### Reanalysis data

The European Centre for Medium-Range Weather Forecasts (ECMWF) European Reanalysis-Interim (ERA-Interim) dataset is used here to calculate severe weather indices (SWI) (Berrisford et al. 2011; Dee et al. 2011). The reanalysis fields used are the air temperature, relative humidity, u and v wind velocity components, geopotential height, relative vorticity, and sea level pressure. Fields are extracted at all pressure levels between 1000 and 100 hPa, as well as at the surface (2 m for temperature and 10 m for winds), and interpolated to a regular, 0.75°, latitude/longitude grid for the period January 1st, 1979 to December 31st, 2015.

Fourteen different SWI were calculated with the equations for each summarized in Table 1. Each parameter is calculated for each 6-h time step between 1979 and 2015 and for all ERA-Interim grid cells over Australia and then averaged over each of the four seasons. Indices are calculated with the SHARPpy open-source sounding and hodograph analysis routines (Blumberg et. al. 2017). Reanalysis pressure levels that were larger than the pressure at the surface were removed from the vertical profiles used for indices calculations to make sure that valid atmospheric profiles were used over higher terrain or synoptically active regimes.

**Table 1:** Severe weather indices (SWI) with variables defined in Appendix 1 (Table 11)

Parameter Name	Abbreviated Name	Formula	References
Bulk richardson shear	brnShr	$\frac{{\sqrt {\left( {\overline{{u_{{6{\text{km}}}} }} - \overline{{u_{{500{\text{m}}}} }} } \right)^{2} + \left( {\overline{{v_{{{\text{6km}}}} }} - \overline{{v_{{500{\text{m}}}} }} } \right)^{2} }^{2} }}{2}$	Rasmussen and Blanchard (1998)
Lifted index	LiMax, Li3, Li5	${\text{T}}_{i} - {\text{T}}_{{{\text{sfc }} \to {\text{i}}}}$ $\left( {{\text{i}} = {\text{max}},{ }\overline{{300{\text{hPa}}}} ,{ }\overline{{500{\text{hPa}}}} } \right)$	Galway (1956)
Most unstable convective available potential energy	muCAPE	${\text{g}}\mathop \smallint \limits_{{{\text{z}}_{{{\text{LFC}}}} }}^{{{\text{z}}_{{{\text{EL}}}} }} \frac{{{\uptheta }_{{{\text{Ep}}}} - {\uptheta }_{{{\text{Ee}}}} }}{{{\uptheta }_{{{\text{Ee}}}} }}{\text{dz}}$	Moncrieff and Miller (1976)
Significant severe	Sig.Sev	${\text{muCAPE*DLS}}$	Brooks et al. (2003b)
Downdraft CAPE	dCAPE	$- \mathop \smallint \limits_{{{\text{p}}_{{{\text{sfc}}}} }}^{{{\text{p}}_{{{\text{LFS}}}} }} \left( {{\upalpha }_{{\text{p}}} - {\upalpha }_{{\text{e}}} } \right){\text{dp}}$	Gilmore and Wicker (1998)
Bulk richardson number	brn	$\frac{{{\text{g}}\mathop \smallint \nolimits_{{{\text{z}}_{{{\text{LFC}}}} }}^{{{\text{z}}_{{{\text{EL}}}} }} \frac{{{\uptheta }_{{{\text{Ep}}}} - {\uptheta }_{{{\text{Ee}}}} }}{{{\uptheta }_{{{\text{Ee}}}} }}{\text{dz}}}}{{0.5* \sqrt {\left( {\overline{{{\text{u}}_{{{\text{6km}}}} }} - \overline{{{\text{u}}_{{{\text{500m}}}} }} } \right)^{2} + \left( {\overline{{{\text{v}}_{{6{\text{km}}}} }} - \overline{{{\text{v}}_{{{\text{500m}}}} }} } \right)^{2} }^{2} }}$	Weisman and Klemp (1982)
Shear	Shr1, Shr3, Shr6	$\sqrt {\left( {u_{i} - u_{{2{\text{m}}}} } \right)^{2} + \left( {v_{i} - v_{{2{\text{m}}}} } \right)^{2} }$ $\left( {{\text{i}} = 1{\text{km}},{ }3{\text{km}},{ }6{\text{km}}} \right)$	–
Convective inhibition	muCIN	$- {\text{g}}\mathop \smallint \limits_{0}^{{{\text{z}}_{{{\text{LFC}}}} }} \frac{{{\uptheta }_{{{\text{Ep}}}} - {\uptheta }_{{{\text{Ee}}}} }}{{{\uptheta }_{{{\text{Ee}}}} }}{\text{dz}}$	Gilmore and Wicker (1998)
Microburst index	MBURST	$$\begin{gathered} \left\{ {\begin{array}{*{20}c} {0\, {\text{if}}\, \theta_{E} \ge 355 } \\ {1\, \theta_{E} < 355} \\ \end{array} } \right\} + \left\{ {\begin{array}{*{20}c} { - 5\, {\text{sbCAPE}} < 2000} \\ {0\, {\text{sbCAPE}} \ge 2000} \\ {1 \,{\text{if}}\, {\text{sbCAPE}} \le 3300} \\ {2\, {\text{sbCAPE}} \ge 3700} \\ {4\, {\text{sbCAPE}} \ge 4300} \\ \end{array} } \right\} \hfill \\ + \left\{ {\begin{array}{*{20}c} {0\, {\text{LI}}_{{{\text{sfc}}}} > - 7.5} \\ {1 \,{\text{if}}\, {\text{LI}}_{{{\text{sfc}}}} \le - 7.5} \\ {2\, {\text{LI}}_{{{\text{sfc}}}} \le - 9 } \\ {3 \,{\text{LI}}_{{{\text{sfc}}}} \le - 10} \\ \end{array} } \right\} \hfill \\ + \left\{ {\begin{array}{*{20}c} {0 \,{\text{ if}}\, \gamma_{{2{\text{m}} - 3{\text{km}}}} \le 8.4 } \\ {1\, \gamma_{{2{\text{m}} - 3{\text{km}}}} > 8.4} \\ \end{array} } \right\} \hfill \\ + \left\{ {\begin{array}{*{20}c} {0 \,{\text{ VT}} < 27} \\ {1\, {\text{if }}\, {\text{VT}} \ge 27} \\ {2 \,{\text{VT}} \ge 28} \\ {3\, {\text{VT}} \ge 29} \\ \end{array} } \right\} \hfill \\ + \left\{ {\begin{array}{*{20}c} {0\, {\text{PWV}} \le 1.7} \\ {0 \, {\text{if}}\, {\text{dCAPE}}\le 900 + {\text{PWV}} \ge 1.7} \\ {1 \,{\text{dCAPE}} > 900 + {\text{PWV}} > 1.7} \\ \end{array} } \right\} + \left\{ {\begin{array}{*{20}c} { - 3 \, {\text{if}} \, {\text{PWV }} \le 1.5} \\ {0 \,{\text{PWV > }}1.5} \\ \end{array} } \right\} + \left\{ {\begin{array}{*{20}c} {1 \,{\text{ if}} \,{\text{TeD }} \ge 35} \\ {0 \,{\text{TeD}} < 35} \\ \end{array} } \right\} \hfill \\ \end{gathered}$$	Blumberg et. al. (2017)
Wind damage parameter	WNDG	$\left( {\frac{{{\text{muCAPE}}}}{2000}} \right)\left( {\frac{{ \gamma_{{2{\text{m}} - 3{\text{km}}}} }}{9}} \right)\left( {\frac{{\overline{{V_{{1 - 3.5{\text{km}}}} }} }}{15}} \right)\left( {\frac{{{\text{muCIN}} + 50}}{40}} \right)$	Blumberg et. al. (2017)

### Bayesian hierarchical model

Bayesian hierarchal modelling is used to determine a relationship between SWI and the rate of convective wind gust occurrence while correcting for biases that result from AWS density influence on the reporting of these events. Hierarchical models allow complex environmental processes to be broken down into a series of conditional models linked together by simple probability rules (Wilke 2003). Such models can be considered from a classical or Bayesian perspective, but Bayesian methods, coupled with Markov Chain Monte Carlo (MCMC) simulation approaches, become necessary when systems are complex (Wilke 2003; Anderson et al. 2007). The freely available Bayesian analysis software WinBUGS (Lunn et al. 2000) is used to develop the hierarchical Bayesian model in this research as previous studies have shown it to be successful when correcting biases in severe weather datasets (Anderson et al. 2007; Cheng et al. 2013, 2016).

The Bayesian hierarchical model developed here is a Binomial-Poisson model that extends what was developed by Cheng et al. (2016) when estimating tornado frequency across North America. The model itself is composed of three sub-models; (1) the observation error model that accounts for non-meteorological factors affecting the fidelity of severe thunderstorm counts in the dataset, (2) the explanatory model that considers the meteorological components causally linked to thunderstorm formation and evolution, and (3) the parameter model that quantifies uncertainty in the parameter values.

#### Observation model

The observation model implemented here follows that of Anderson et al. (2007) and Cheng et al. (2016). The model first specifies a binomial distribution for the reported number of events, Eobs_i_, for a given area or grid cell, i, and time period, conditional on the actual (but unobserved) event occurrences, Elatent_i_, for the same area and time period. The binomial distribution is a discrete distribution (Bain and Engelhardt 1992) that is commonly used where Eobs_i_ can be thought of as the number of “successes” in Elatent_i_ independent Bernoulli trails, with p_i_(${\upalpha }_{{{\text{StationD}}}} ,\;{\upbeta }_{{{\text{StationD}}}}$), the probability of detecting an event for the given density effect parameters, ${\upalpha }_{{{\text{StationD}}}} ,\;{\upbeta }_{{{\text{StationD}}}}$, where${\text{StationD}}$ represents the time averaged AWS density, as defined in Sect. 2.1. This relationship is formally written:

1 $$\left( {\left. {{\text{Eobs}}_{{\text{i}}} } \right|{\text{Elatent}}_{{\text{i}}} ,{\uplambda }_{{\text{i}}} ,{\text{p}}_{{\text{i}}} \left( {{\upalpha }_{{{\text{StationD}}}} ,\;{\upbeta }_{{{\text{StationD}}}} } \right)} \right)\sim {\text{Binomial}}\left[ {{\text{Elatent}}_{{\text{i}}} {\text{p}}_{{\text{i}}} \left( {{\upalpha }_{{{\text{StationD}}}} ,\;{\upbeta }_{{{\text{StationD}}}} } \right)} \right],$$

where ${\uplambda }_{{\text{i}}}$, is the expected event frequency. The probability of detecting a convective wind gust in a given cell, should it occur, p_i_, is a function of ${\text{StationD}}_{{\text{i}}}$. Cheng et al. (2016) defined this relationship using an exponent model but for population density. However, preliminary tests found the use of their formulation with station density in place of population density led to unrealistic values. Using the sparse network of AWS in Australia, which can only observe at a given point, instead of population density make it more likely for small-scale convective wind events to pass between stations and not be recorded in the observational record. This could impact the accuracy of the probability of detection relationship used. As such a modified version of their exponent model was developed:

2 $${\text{p}}_{{\text{i}}} \left( {{\upalpha }_{{{\text{StationD}}}} ,{\upbeta }_{{{\text{StationD}}}} } \right) = 1 - {\text{exp}}\left( { - 1{*}\left( {\frac{{{\text{StationD}}_{{\text{i}}} }}{{{\upalpha }_{{{\text{StationD}}}} }}} \right)^{{{\upbeta }_{{{\text{StationD}}}} }} } \right),$$

where ${\text{stationD}}_{{\text{i}}}$ is the station density of the ith area, ${\upbeta }_{{{\text{StationD}}}}$ is a parameter that controls the shape of the *p*_i_ curve, and ${\upalpha }_{{{\text{StationD}}}}$ determines the slope of *p*_i_. The value of ${\upalpha }_{{{\text{StationD}}}}$ is constrained between 0.0027 and 0.000356 to help ensure the model finds a physically realistic p_i_ curve. While constraining ${\upalpha }_{{{\text{StationD}}}}$ in this manner does, to some degree, limit the ability of the model to freely develop a probability of detection relationship, adding some constraint to the probability of detection has been shown necessary in previous work using Bayesian Hierarchical Modelling (Potvin et al. 2019; 2022). Equation 2 results in a *p*_i_ of 1 for large ${\text{Station}}\;{\text{D}}_{{\text{i}}} { }$ and approaches zero for a small or zero ${\text{StationD}}_{{\text{i}}}$.

The final component to the observational sub-model is to account for the chance of irregular spatial variation within each ith area due to irregular station distribution as well as to account for the randomness at which convective wind gusts occur. A Poisson process counts the occurrence of events at a certain rate with random occurrence. Therefore, the true climatological event count, Elatent_i_, is modelled as a Poisson process (Bain and Engelhardt 1992) conditioned on a climatological event frequency per cell, $\lambda_{i}$, since the true climatological event count can often depart for the climatological event frequency due to the randomness of these events:

3 $$\left( {\left. {{\text{Elatent}}_{i} } \right|\lambda_{i} } \right)\sim {\text{Poisson}}\left( {\lambda_{i} } \right).$$

Elatent_i_ is bounded by zero and will, by definition, be greater or equal to Eobs_i_. While the Binomial-Poisson model was chosen for the Bayesian hierarchical model in this work other models could prove beneficial as well, such as the Zero-Inflated Poisson model (e.g. Cheng et al. 2015; 2016) or the Negative-Binomial model (e.g. Elsner and Widen 2014; Elsner et al. 2016), all three types of models account for overdispersion.

#### Explanatory model

The explanatory model is the logarithm of the expected event frequency, λ_i_, as a linear function of several explanatory variables, x:

4 $$\ln (\lambda_{i} ) = \alpha_{0} + \alpha_{1} x_{i1} + \alpha_{2} x_{i2} + \ldots + \alpha_{k} x_{ik} + {\text{CAR}}_{i} ,$$

where ${\text{x}}_{{{\text{ik}}}} { }$ is the value of the kth explanatory variable (e.g. muCAPE, muCIN, etc.) for the ith grid cell and ${\upalpha }_{{\text{k}}}$ are regression coefficients corresponding to the kth explanatory variable with ${\upalpha }_{0}$ the intercept. In addition to the explanatory variables an additional, conditional autoregressive term, ${\text{CAR}}_{{\text{i}}}$, is added to Eq. 4. The ${\text{CAR}}_{{\text{i}}}$ term is a grid cell specific, random effect term that is designed to capture variability of severe convective gust occurrence that is not explicitly considered by the explanatory variables (Cheng et al. 2016). Examples of this may include orographic influence on the formation of thunderstorms (Taylor et al. 2011) or possible lake or sea-breeze convergence-influenced convective processes (King et al. 2003). Given this, Cheng et al. (2016) assume the ${\text{CAR}}_{{\text{i}}}$ term to have regional characteristics and therefore to be spatially correlated. This term is based on the Bayesian conditional autoregressive model (Besag et al. 1991) where each grid cell term is jointly distributed as a multi-variate normal distribution with zero mean and an unknown covariance matrix (Besag and Kooperberg 1995). As implemented here, the model assumes each ${\text{CAR}}_{{\text{i}}}$ depends only on the neighbouring cells and that all neighbours have equal influence (weight of 1). The term is therefore defined as:

5 $${\text{CAR}}_{{\text{i}}} { }\sim {\text{ Normal}}\left( {{\upmu }_{{\text{i}}} ,\frac{{{\upsigma }^{2} }}{{{\text{n}}_{{\text{i}}} }}} \right),$$

where

6 $$\mu_{i} = \frac{1}{{n_{i} }}\mathop \sum \limits_{{j \in N_{i} }} {\text{CAR}}_{j}$$

with $n_{i}$ being the number of adjacent grid cells, *j* refers to the adjacent cells, and $N_{i}$ is the n^th^ adjacent cell.

In this research, models that use 1, 2, or 3 explanatory variables within Eq. 4 to determine which combination of explanatory variables best explain the expected event frequency, *λ*_*i*_, across all cells will be tested (Table 2). The explanatory models with 1 variable mostly utilize composite indices, i.e. those that include multiple measures of the state of the atmosphere into a single index. The 2 indices models incorporate an instability index with a shear index. Finally, the 3 variable models will look at an instability index in combination with a shear index and one additional explanatory variable.

**Table 2:** List of SWI combinations considered

	1st Variable (One of listed indices)	2nd Variable (One of listed indices)	3rd Variable (One of listed indices)
1 Index models	Most Unstable CAPE Downdraft CAPE Microburst Index Wind Damage Parameter Significant Severe 300 hPa Lifted Index 500 hPa Lifted Index Maximum Lifted Index	–	–
2 Indices models	Most Unstable CAPE 300 hPa Lifted Index 500 hPa Lifted Index Maximum Lifted Index	0 to 1 km Shear 0 to 3 km Shear 0 to 6 km Shear Bulk Richardson Shear	–
3 Indices models	Most Unstable CAPE 300 hPa Lifted Index 500 hPa Lifted Index Maximum Lifted Index	0 to 1 km Shear 0 to 3 km Shear 0 to 6 km Shear Bulk Richardson Shear	Downdraft CAPE Microburst Index Wind Damage Parameter CIN

Given each index has their own measurement scales, all indices are standardized prior to their use in Eq. 4 (i.e. x_ik_). Similar to Cheng et al. (2015), this standardization is done using the z-score function in MATLAB and therefore is a representation of how many standard deviations a given index is above or below the mean seasonal index calculated across Australia over the 2005–2015 period.

#### Parameter model

The parameter model treats each parameter as a random variable rather than a fixed quantity (Anderson et al. 2007). The parameters ${\upbeta }_{{{\text{StationD}}}} { }$ and ${\upalpha }_{{\text{k}}}$ are estimated using the Bayesian approach of assigning prior distributions, which are assumed here to be non-informative by making them “flat”. The parameter, ${\upalpha }_{{{\text{StationD}}}}$, is also assigned a “flat” prior distribution but is constrained between 0.0027 and 0.000356. The model will then determine the shape of the posterior distributions. The regression coefficients, ${\upalpha }_{{\text{k}}}$, are assigned a normal distribution (Bain and Engelhardt 1992) with a mean of 0 and variance of 10,000 (implying that the prior distribution is non-informative). Similarly, the density effect parameters, ${\upalpha }_{{{\text{StationD}}}} ,{\upbeta }_{{{\text{StationD}}}}$, are assigned a normal distribution with a mean of 1 and variance of 10,000. These are formally shown in Eqs. 7 – 10.

7 $${\upalpha }_{{{\text{StationD}}}} { }\sim {\text{ Normal}}\left( {1,{ }10{ }000} \right)){\text{I}}\left( {0.0027,{ }0.000356} \right),$$

8 $${\upbeta }_{{{\text{StationD}}}} { }\sim {\text{ Normal}}\left( {1,{ }10{ }000} \right),$$

9 $${\upalpha }_{{\text{k}}} { }\sim {\text{ Normal}}\left( {0,{ }10{ }000} \right),$$

10 $${\upsigma }^{2} { }\sim {\text{ Inverse Gamma}}\left( {0.01,{ }0.01} \right),$$

#### Model implementation

The Bayesian model is run using 73 different combinations of the 14 variables listed in Table 1, spread across the 1, 2, and 3 parameter implementations. The distribution and mean of all parameters, regression coefficients, and resulting expected event frequency discussed in Sects. 2.3.1–2.3.3 are calculated for each of the 4 seasons using the period from 2005 to 2015. Each model starts with two different initialization points and results in output of two Markov chain Monte-Carlo (MCMC) “chains”, which are used to test for convergence of the model as well as the model performance (Cheng et al. 2013, 2015, 2016). For each model run, the first 5,000 iteration are discarded (burn in) to remove any initial model start-up instabilities prior to running a further 50,000 iterations to allow the models to converge. The final 7,500 iterations are kept for analysis with the model convergence for each season assessed. The methods used for testing convergence are discussed in Sect. 2.4.

### Model performance

The first step in selecting the optimal combinations of indices for use in Eq. 4 is to ensure they result in convergence of the Bayesian model. Convergence is checked using the Gelman-Rubin convergence diagnostic (Gelman and Rubin 1992; Brooks and Gelman 1998), which is based on analysis of the “chain” (i.e. the time series) of each parameter when started from two different initialization points. This convergence diagnostic works by comparing the estimated variance between chains, how similar the chains are to each other, and the variance within chains (mean variance of each chain) for each model parameter. Brooks and Gelman (1998) suggest a Gelman-Rubin score of less than 1.2 is required for all model parameters and regression coefficients (i.e. ${\upalpha }_{0}$, ${\upalpha }_{1}$, ${\upalpha }_{2} ,$ ${\upalpha }_{3}$, ${\upalpha }_{{{\text{StationD}}}}$, ${\upbeta }_{{{\text{StationD}}}}$, σ) to be confident that convergence has occurred. The models found not to meet this criterion were discarded and further analysis conducted only on the remaining set of models.

The second step uses the model parameters and regression coefficients outputs, from the remaining set of models, to *estimate* the number of days with at least one convective gust event observed per cell (Eobs_i_) for each year from 2005 to 2015, where Eobs_iy_ will represent the *actual* number of event days observed in a given cell, i, for a given year, y, and EobsM_iy_ will represent the *model estimated* of this value. For each of the “converged” models, EobsM_iy_ is calculated in three steps. (1) the expected event frequency for a given year, λ_iy_, is calculated from Eq. 4 using a Monte-Carlo simulation with a sample size of 20,000. Since the Bayesian models output the parameters and regression coefficients as a distribution, running the Monte-Carlo simulation is necessary. In contrast to the methods discussed in Sect. 2.3, the explanatory variables now use the mean seasonal values for the given year, not the period 2005–2015. The regression coefficients (${\upalpha }_{0}$, ${\upalpha }_{1}$, ${\upalpha }_{2} ,$ ${\upalpha }_{3}$) use the distributions determined by the trained models. (2) Elatent_iy_ for the given year is then solved by sampling from the Poisson distribution of ${\uplambda }_{{{\text{iy}}}}$. Similarly, the distributions for ${\upalpha }_{{{\text{StationD}}}} { }$ and ${\upbeta }_{{{\text{StationD}}}}$ are randomly sampled to solve for the probability of detecting an event, p_iy_, of each grid-cell for the given year. (3) EobsM_iy_ is then determine by taking the inverse binomial of p_iy_ and Elatent_iy_.

Two metrics were used to test the performance of all models that “converged”. They are, the Mean Absolute Error (MAE) and the total conditional autoregressive (Total CAR) term. The MAE is calculated by taking the absolute error between the EobsM_iy_ value for each cell and the corresponding Eobs_iy_ for the same cell and year. The absolute error is then averaged over the 11-year period for all ERA-Interim cells that have AWS. The sum of the absolute value of the CAR terms over all cells is the Total CAR value. Since the CAR term accounts for things not accounted for by the explanatory variables within the Bayesian model the Total CAR provides an indication of how well the explanatory variables can explain $\lambda_{i}$. A small Total CAR suggests they explain $\lambda_{i}$ well while a large Total CAR suggest they do so poorly.

Identifying suitable model parameter combinations using multiple performance metrics is not straightforward. A ranking scheme for doing this is used here. First, the models are ranked based on MAE. To help compare the performance between models the difference between each model and the model with the minimum MAE are calculated. This so-called percent difference, $\Delta \varepsilon$, when calculated for MAE or Total CAR, is defined as:

11 $$\Delta \varepsilon_{{\text{E}}} = { }100{*}\left( {\frac{{\varepsilon_{{{\text{E}},{\text{k}}}} }}{{\varepsilon_{{{\text{E}},1}} }} - 1} \right)$$

where $\varepsilon_{E}$ is the error term for one of the two metrics, E, (i.e. *ε*_*t*CAR,_ ε_MAE_), *k* is the *k*th combination being assessed, and $\varepsilon_{E,1}$ indicates the first ranked (i.e. lowest error) model error value. All these models were then individually assessed to ensure that the coefficient distributions and $\alpha_{{{\text{StationD}}}}$, $\beta_{{{\text{StationD}}}} { }$ terms converged for both chains as assessed using the Gelman-Ruben score. In addition, the values assigned to the coefficient of each of the indices for each model were checked to ensure they were consistent with the physical understanding of the index and how it related to the occurrence of severe convection.

## Results and discussion

### Model comparisons

In this section, the models shown to converge are listed and sorted from smallest MAE to largest. The top 5 models are retained for the discussion. This is done for each season. The model performance metrics as well as the mean and standard deviation values of the model coefficient are discussed in Sections, 3.1.1–3.1.4. The outputs from the models chosen in Sects. 3.1.1–3.1.4 for each season are then analysed in Sect. 3.3.

#### Autumn

For Autumn (March to May), only 13 of the 73 models converged when considering their Gelman-Ruben score. Results for the top 5 models are shown in Table 3, with their corresponding Total CAR, MAE, and percent differences values listed. There is a mix of 1, 2, and 3 indices models that met the convergence criteria set out in Sect. 2.3. However, there are only 4 models where ΔεMAE is less than 20%. These are the 500 hPa Lifted Index and 1 km Shear model (Li5-Shr1), the 300 hPa Lifted Index model (Li3), 500 hPa Lifted Index and Bulk Richardson Shear model (Li5-brnShr), and the Most Unstable CAPE and 6 km Shear model (muCAPE-Shr6). The Li5-Shr1 model has both the smallest Total CAR and MAE.

**Table 3:** List of the top 5 models for Autumn and their associated Total CAR and mean absolute error (MAE)

Model	MAE (event days)	Total CAR	Δ*εt*CAR (%)	Δ*ε*MAE (%)
Li5-Shr1	**0.15**	**125.82**	–	–
Li3	0.18	298.24	137.03	17.90
Li5-brnShr	0.18	275.49	118.96	18.20
muCAPE-Shr6	0.18	282.10	124.21	19.55
Li5-Shr6	0.21	369.28	193.50	41.38

Examining the coefficient values output by the Bayesian models allows an assessment of whether the model is generating coefficients that could be considered physically reasonable, given the atmospheric processes they are representing. Since the Bayesian models output a distribution for the coefficients the mean and standard deviation of the coefficients for the models listed in Table 3 are shown in Table 4. For the Li5-Shr1 model, the instability index, Li5, has a small negative coefficient ($\alpha_{1}$) value of − 0.29. This intuitively makes sense as larger negative values of the lifted index represents a more unstable atmosphere and therefore would suggest more likely occurrence of convective events. The shear index, Shr1, also has a small negative mean coefficient ($\alpha_{2}$) of − 0.48, which is somewhat counterintuitive as this suggests environments with shr1 values less than the mean (as measured across all Australia for that season) are more conducive to gust occurrence than those with higher shear, as would typically be expected (Klemp 1987; Markowski and Richarson 2009, 2013). However, Westermayer et al. (2017) show that convection can occur in both strong and weak shear environments, with results here suggesting that during the Autumn months there is a preference for the weaker shear environments. The Li5-Shr1 model is used to develop the Autumn climatology shown in Sect. 3.3 since it is shown to converge and has small values for both CAR Total and MAE.

**Table 4:** Mean and standard deviation of coefficient values for the five best performing autumn models

Model	${\upalpha }_{0}$ Mean	${\upalpha }_{0}$ StdDev	${\upalpha }_{1}$ Mean	${\upalpha }_{1}$ StdDev	${\upalpha }_{2}$ Mean	${\upalpha }_{2}$ StdDev	${\upalpha }_{3}$ Mean	${\upalpha }_{3}$ StdDev
Li5-Shr1	**1.15**	**0.48**	**− 0.29**	**0.19**	**− 0.48**	**0.18**	–	–
Li3	1.08	0.67	− 0.13	0.29	–	–	–	–
Li5-brnShr	1.04	0.59	− 0.23	0.29	0.14	0.20	–	–
muCAPE-Shr6	1.02	0.59	− 0.01	0.32	− 0.13	0.27	–	–
Li5-Shr6	0.97	0.57	− 0.10	0.48	− 0.10	0.37	–	–

#### Winter

Only 6 models converged during the winter months (June–August) when considering their Gelman-Ruben score. This small number is primarily a result of the low observed event counts during these months, which makes it challenging to converge to a solution. Another possible cause may be that the typical indices used to explain or predict the occurrence of severe weather may not be entirely appropriate in the winter months. More tailored indices for the kind of convective events that occur in the winter months, such as cool-season tornadoes (Kounkou et al. 2009; Hanstrum et al. 2002), may need to be developed. Irrespective, of the 6 models that converged, the LiMax-Shr3 stood out as the best performing when examining the Total CAR, and MAE. All other models generated MAE values greater than double that of the LiMax-Shr3 model and Total CAR values greater than ten times its value. Interestingly, no three-index models were found to converge for winter. Table 5 shows the value of the error metrics for the top 5 winter models.

**Table 5:** As for Table 3 but for winter

Model	MAE (event days)	Total CAR	Δ*εt*CAR (%)	Δ*ε*MAE (%)
LiMax-Shr3	**0.56**	**50.07**	–	–
muCAPE-Shr1	1.13	672.40	1242.83	101.29
muCAPE-Shr6	1.37	738.51	1374.87	143.89
Mburst	1.95	795.14	1487.96	247.19
Li5-brnShr	3.50	836.96	1571.48	524.12

The coefficient values for the LiMax-Shr3 model are shown in Table 6. This model has a negative value for the mean $\alpha_{0}$ coefficient which, recalling that the natural logarithm of the expected event frequency *λ*_*i*_ is used in Eq. 4, is an indication of a low number of expected events during the winter months. Mean $\alpha_{1}$ and $\alpha_{2}$ coefficients, − 0.52 and 0.90, respectively, also make physical sense. That is ${\upalpha }_{1}$ is negative and again means larger negative values of the index (LiMax) signifies a more unstable atmosphere will increase the number of event occurrences. The mean coefficient ($\alpha_{2}$) for the shear index (Shr3) being positive also accords with physical reasoning (and the discussion of shr1 in Sect. 3.1.1), with regions with higher shear values typically exhibiting greater numbers of events. Since the LiMax-Shr3 model is shown to have converged, performed significantly better than all other models considered for this work, and has coefficients that accord with physical reasoning, it is used for the final climatology, shown in Sect. 3.3, during winter months.

**Table 6:** As for Table 4 but for winter

Model	${\upalpha }_{0}$ Mean	${\upalpha }_{0} { }$ StdDev	${\upalpha }_{1}$ Mean	${\upalpha }_{1}$ StdDev	${\upalpha }_{2}$ Mean	${\upalpha }_{2}$ StdDev	${\upalpha }_{3}$ Mean	${\upalpha }_{3}$ StdDev
LiMax-Shr3	**− 1.08**	**0.71**	**− 0.52**	**0.02**	**0.90**	**0.37**	–	–
muCAPE-Shr6	− 0.64	0.96	− 0.12	0.42	0.30	0.31	–	–
muCAPE-Shr6	− 1.53	1.05	− 1.03	0.65	1.53	0.95	–	–
Mburst	− 1.87	1.23	− 1.42	0.77	–	–	–	–
Li5-brnShr	− 1.04	0.98	0.67	0.46	− 0.05	0.22	–	–

#### Spring

For Spring (September to November) 29 of the 73 models were found to convergence based on the Gelman-Ruben criteria with the top 5 shown in Table 7. Out of these models, 11 models have MAE values within 20% of the smallest MAE, suggesting a similar performance for a wide range of index combinations. However, while the LiMax model has the smallest MAE it also has the largest Total CAR out of the 5 models shown in Table 7, which is 50% higher than the model producing the smallest Total CAR (LiMax-Shr6-dCAPE – not shown in table). The model that generates the smallest Total CAR is the single index Mburst model which has the second smallest MAE.

**Table 7:** As for Table 3 but for spring

Model	MAE (event days)	Total CAR	Δ*εt*CAR (%)	Δ*ε*MAE (%)
LiMax	0.30	452.10	50.03	–
Mburst	**0.31**	**393.55**	**30.60**	**4.20**
Li5-Shr1	0.31	411.92	36.70	5.20
Li5	0.32	400.70	32.97	6.70
muCAPE-Shr1	0.32	424.83	40.98	7.28

The coefficients for each of the 5 models with lowest MAE are given in Table 8. The $\alpha_{0}$ value are all shown to approach 2, which signifies larger base levels of event frequency during spring than observed for autumn or winter. The mean coefficient ($\alpha_{1}$) for Mburst, of 0.06, is shown to be positive, which is reasonable given larger magnitudes of the composite index (Table 1) are indicative of higher potential for microburst occurrence (Blumberg et. al. 2017). Another model of interest is the Li5-Shr1 model, which was also discussed in Sect. 3.1.1, for the autumn months. For the spring months, this model has coefficients of -0.06 for Li5 and -0.05 for Shr1, the same signs as seen for the autumn version of this model. Finding similar coefficients for the low level shear indices, during different seasons, shows consistency in how the Bayesian model tries to related environments to severe convective wind gust occurrences and may highlight that the relationship between low level shear and these events may not be a simple linear relationship. Given the smaller Total CAR and MAE, the Mburst model is used to develop the Spring climatology shown in Sect. 3.3.

**Table 8:** As for Table 4 but for spring

Model	${\upalpha }_{0}$ Mean	${\upalpha }_{0} { }$ StdDev	${\upalpha }_{1}$ Mean	${\upalpha }_{1}$ StdDev	${\upalpha }_{2}$ Mean	${\upalpha }_{2}$ StdDev	${\upalpha }_{3}$ Mean	${\upalpha }_{3}$ StdDev
LiMax	1.77	0.51	0.08	0.20	–	–	–	–
Mburst	**1.88**	**0.45**	**0.06**	**0.25**	–	–	–	–
Li5-Shr1	1.89	0.45	− 0.06	0.23	− 0.05	0.15	–	–
Li5	1.92	0.46	− 0.10	0.25	–	–	–	–
muCAPE-Shr1	1.90	0.43	− 0.01	0.26	− 0.06	0.16	–	–

#### Summer

Lastly, summer (December to February) is examined. Six models were found to converge for summer according to the Gelman-Ruben criteria with the top 5 models shown in Table 9. Of these, 3 models have an MAE within 20% of the lowest value, including the 300 hPa Lifted Index (Li3) and 500 hPa Lifted Index (Li5) single variate models, and a two index model using the most unstable CAPE and 1 km Shear model (muCAPE-Shr1). While these models have similar MAE, it is the muCAPE-Shr1 model that exhibits the smallest Total CAR of the three. The three index Li3-Shr6-WNDG model was also shown to generate a small total CAR but had marginally higher MAE than the three previous models.

**Table 9:** As for Table 3 but for summer

Model	MAE (event days)	Total CAR	Δ*εt*CAR (%)	Δ*ε*MAE (%)
Li3	0.59	760.73	16.07	–
Li5	0.65	722.85	10.29	10.58
MuCAPE-Shr1	**0.67**	**689.71**	**5.24**	**14.28**
Li3-Shr6-WNDG	0.75	668.92	2.06	26.59
Li3-Shr3	0.81	655.40	0.00	37.40

The coefficients for the top 5 summer models are shown in Table 10. All have an $\alpha_{0}$ value great than 2, indicating an even higher base level of event occurrence than in Spring. For the muCAPE-Shr1 model, the coefficient $(\alpha_{1}$) for the instability index, muCAPE, is shown to be positive, 0.67. Given that larger values of muCAPE generally suggest higher levels of instability and higher probability of severe convection, this is reasonably expected. As with the autumn and spring models that incorporate Shr1, the coefficient ($\alpha_{2}$) for Shr1 is negative, -0.19. Again, this was not intuitively expected but the prevalence of convective wind gusts in relatively low shear environments during the warmer months is supported by similar observations in Europe by Pacey et al. (2021). Houston and Wilhelmson (2011) also suggest that deep cold pools may help produce long lived thunderstorms even with small values of vertical shear, which they found to occur when multiple deep convective cells are initialized in close proximity to each other. This follows the observation of long-lived quasi-linear systems in environments with little to no shear (e.g. Fovell and Ogura 1989; Coniglio and Stensrud 2001; Evans and Doswell 2001; Weisman and Rotunno 2004). For the three index Li3-Shr6-WNDG model the coefficients $\alpha_{1}$, $\alpha_{2}$, and $\alpha_{3}$ are 0.11, − 0.64, and 0.01. While we see a similar negative coefficient for its shear parameter, this time using Shr6 instead of Shr1, its instability coefficient for Li3 is positive, suggesting a more stable environment is conducive to severe convective wind gusts occurrence. However, there does appear to be coupling between Li3 and SHR6 that is contributing to this observation. As such, the muCAPE-Shr1 model is used for the summer climatology shown in Sect. 3.3.

**Table 10:** As for Table 4 but for summer

Model	${\upalpha }_{0}$ Mean	${\upalpha }_{0}$ StdDev	${\upalpha }_{1}$ Mean	${\upalpha }_{1}$ StdDev	${\upalpha }_{2}$ Mean	${\upalpha }_{2}$ StdDev	${\upalpha }_{3}$ Mean	${\upalpha }_{3}$ StdDev
Li3	2.61	0.40	− 0.43	0.25	–	–	–	–
Li5	2.68	0.41	**− **0.52	0.28	–	–	–	–
MuCAPE-Shr1	**2.65**	**0.40**	**0.47**	**0.26**	**− 0.19**	**0.16**	–	–
Li3-Shr6-WNDG	2.57	0.46	0.11	0.34	− 0.64	0.30	0.01	0.24
Li3-Shr3	2.92	0.42	− 0.31	0.25	− 0.51	0.17	–	–

### Probability of detection

**Fig. 3:**
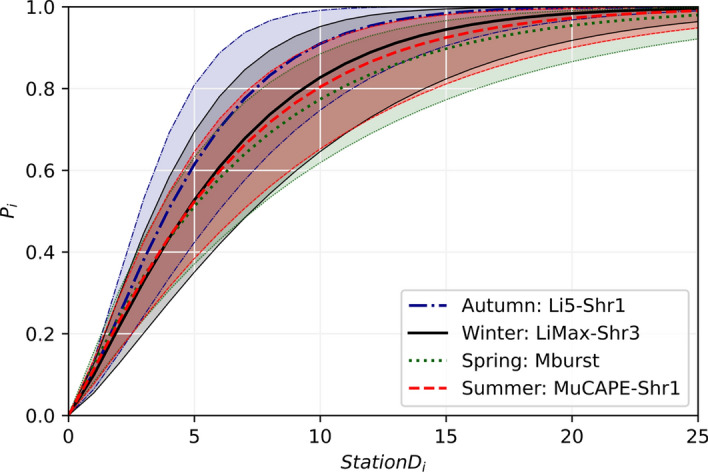
Probability of detection ($p_{i}$) curves for each of the seasons along with their corresponding 25th and 75th percentile curves

Using the models identified in Sect. 3.1 for each of the four seasons, the mean calculated Probability of Detection ($p_{i}$) relationship (Eq. 2) is plotted in Fig. 3 as a function of the number of AWS per grid cell, StationD_i_. Each curve generally follows the same shape exhibiting a relatively sharp increase in $p_{i}$ until the StationD_i_ increases to around ten stations per cell, after which $p_{i}$ level off as it asymptotes to one. The point at which $p_{i}$ reaches 0.99 varies for each model/season, with AWS counts per cell of 18, 23, 32, and 27 found for the autumn, winter, spring, and summer months, respectively. It is not immediately clear why the $p_{i}$ curves would vary for the different seasons, and for the most part the $p_{i}$ curves fall within the 50% credible intervals for all other seasons. However, it is hypothesized here that differences in convective outflow characteristics (e.g. size and duration) during different periods of the year might impact the AWS density required to observe them. One could reason that larger convective system (e.g. linear convective systems) would be more likely observed by the AWS than more isolated convective system (e.g. multicellular, supercells) and thus require a less dense network of AWS to detect them. There may be a seasonal link driving this, for example, during the autumn and winter months Australia experiences more extratropical cyclones and their associated fronts which may initiate more large-scale convective events. Overall, we find that while each model determines a slightly different relationship between $p_{i}$ and StationsD_i_, they all follow the same general shape and trend, particularly during the Spring and Summer months when event numbers are most significant.

### Australian convective wind gust climatology

Using the chosen seasonal models, a spatially complete seasonal convective wind gust climatology is developed for autumn (Li5-Shr1), winter (LiMax-Shr3), spring (Mburst), and summer (MuCAPE-Shr1). Seasonal climatologies based on the period 2005–2015 (i.e. the period where observational data is available) are shown and discussed in Sect. 3.3.1. However, given it is possible to apply these models, once trained, over periods where observational data does not exist, Sect. 3.3.2 discusses their application to an extended ERA-Interim reanalysis period ranging from 1979 to 2015. This allows for a characterization of the convective gust climate over a 37-year period, which is deemed more appropriate for assessing the true climate.

#### 2005–2015 climatology

**Fig. 4:**
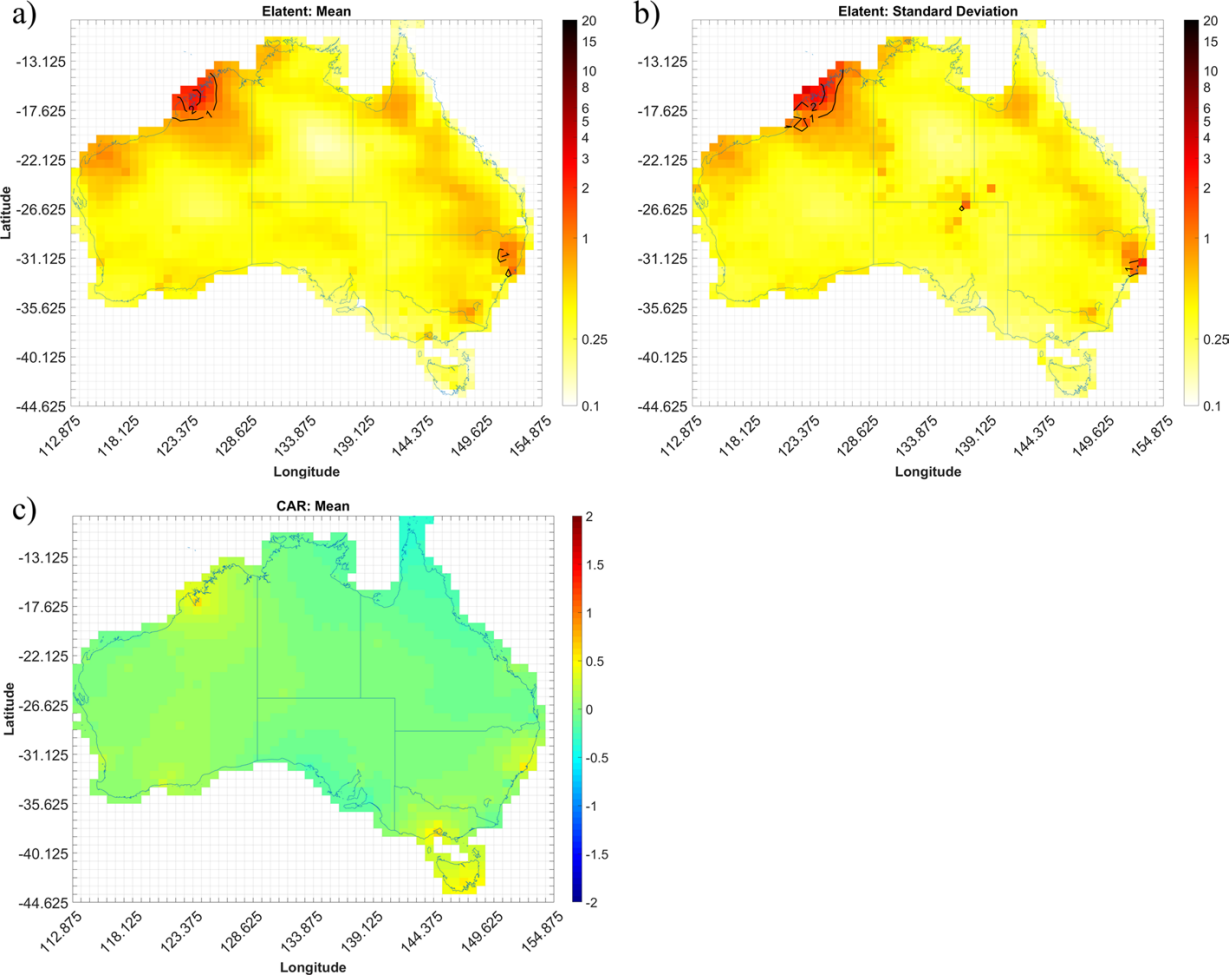
Output from the Li5-Shr1 autumn model for the period 2005–2015; **a** annualized mean gust event count (Elatent), **b** standard deviation of Elatent, **c** mean conditional autoregressive (CAR) term, per grid cell (0.75 X 0.75°)

**Fig. 5:**
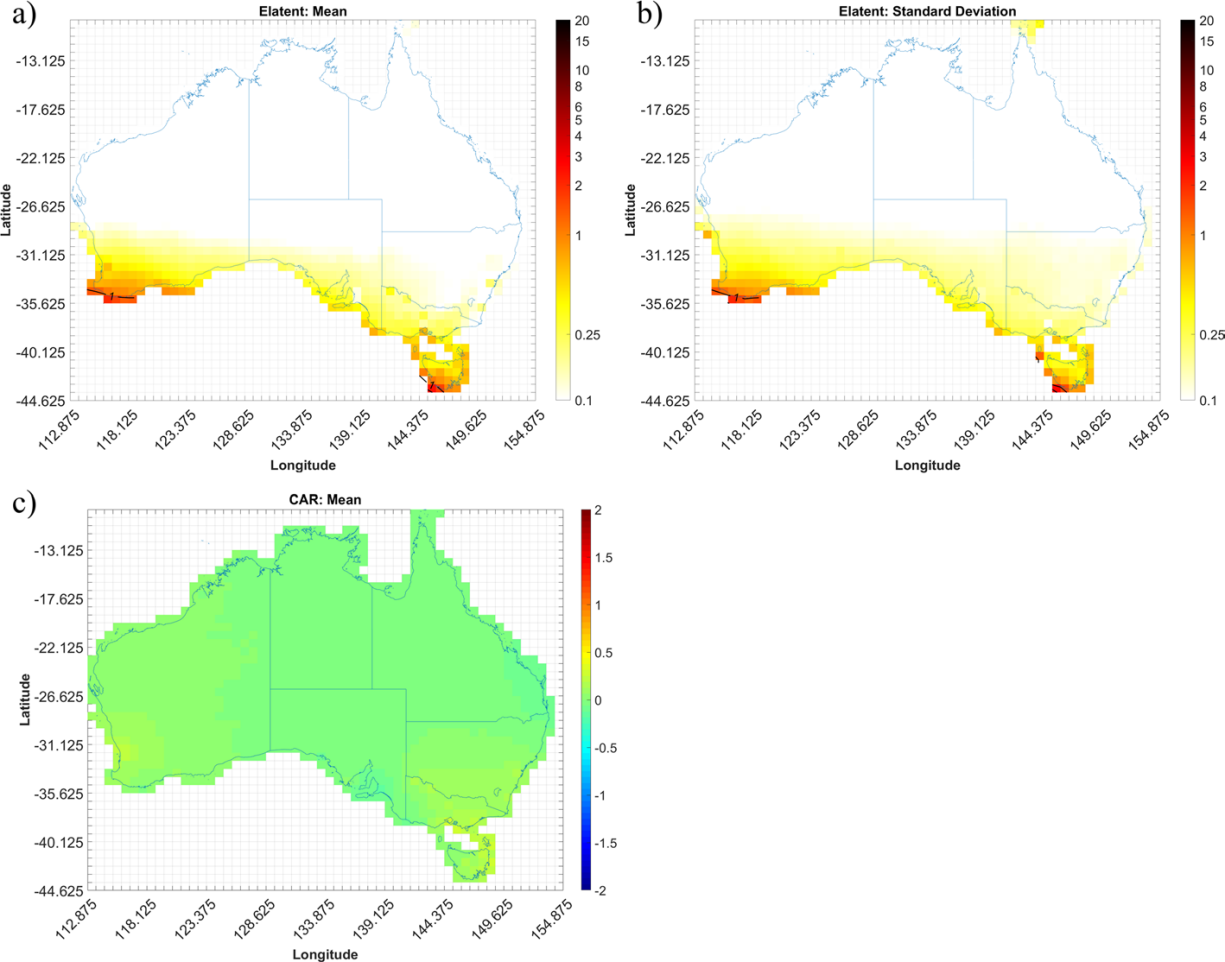
As for Fig. 4 but showing LiMax-Shr3 winter model

**Fig. 6:**
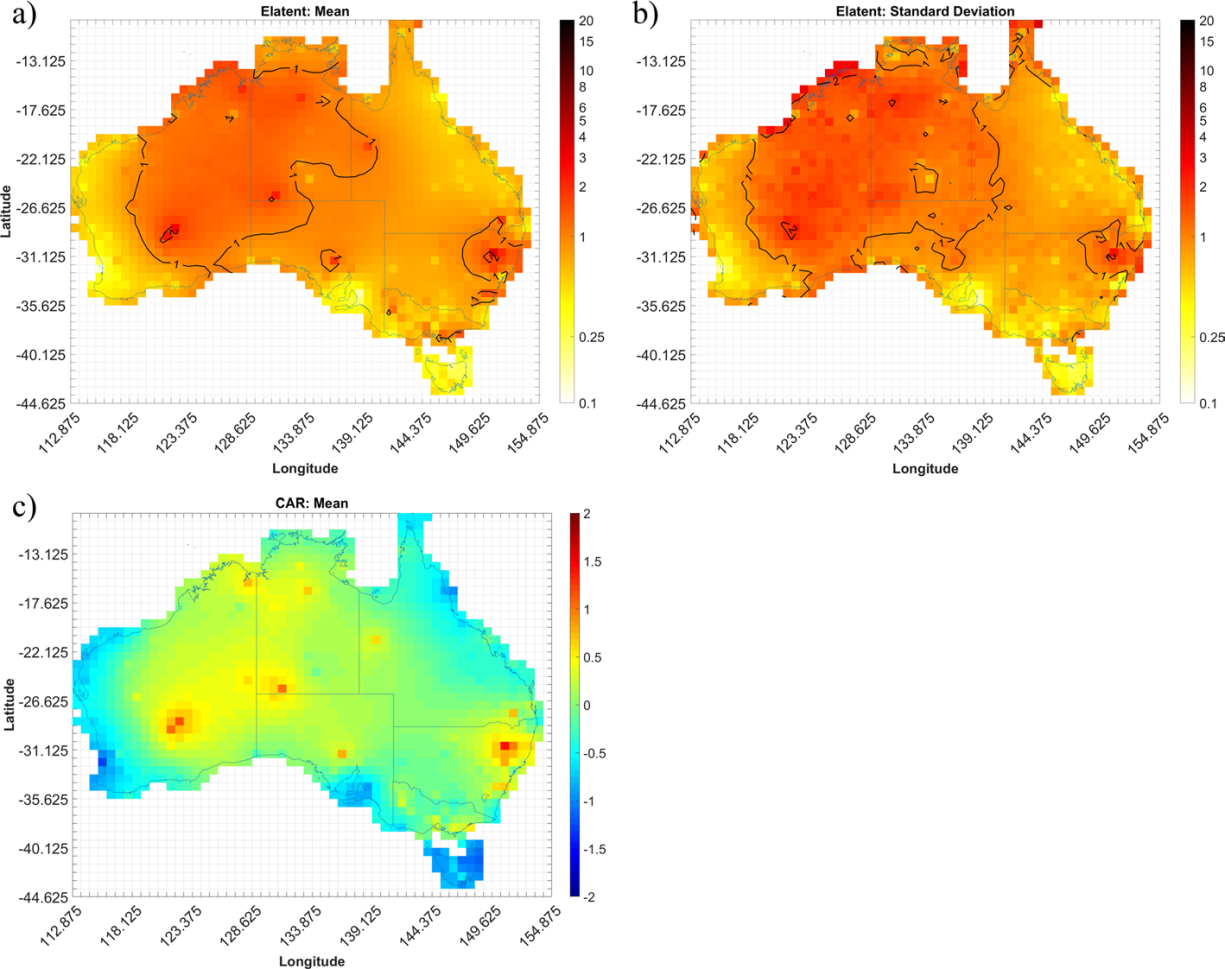
As for Fig. 4 but showing Mburst spring model

**Fig. 7:**
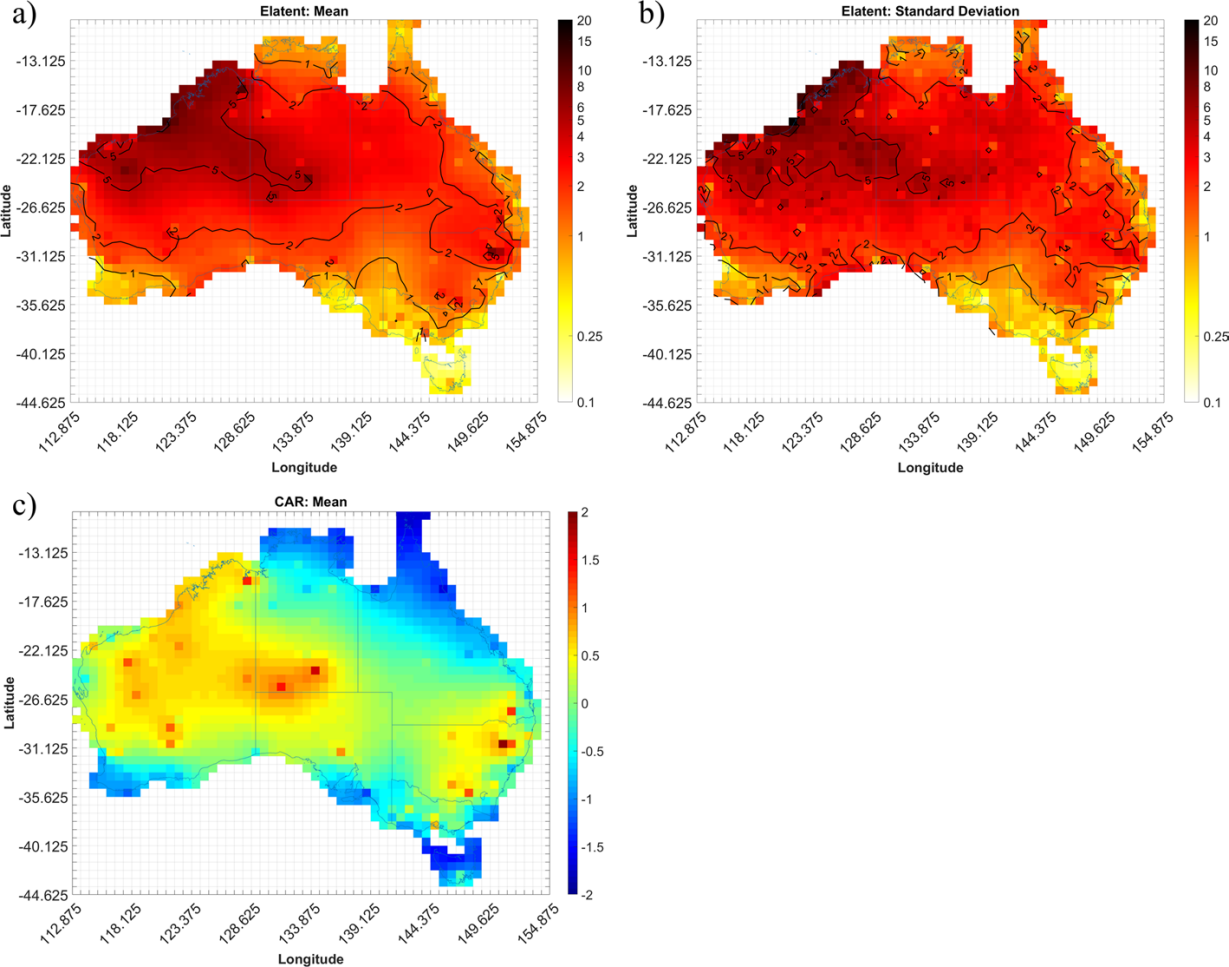
As for Fig. 4 but showing muCAPE-Shr1 summer model

The expected number of convective gust events (Elatent) per season for each grid cell across Australia are shown in Figs. 4, 5, 6 and 7. Annualized mean and standard deviations, as given by the Bayesian models, of the possible number of events over the entire period are shown, as well as the mean conditional autoregressive term (CAR) term. Results for autumn, winter, spring, and summer are shown in Figs. 4, 5, 6 and 7, respectively.

Figure 4a shows that the Li5-Shr1 model estimates there to be, on average, no more than 2 days that experience a convective gust event within any given grid cell during autumn each year. A peak in events is shown to occur in the northern half of Western Australia (WA), with a particular “hot spot” along the northern coast. The model suggests less than 0.5 event days (i.e. one day where a severe wind storm occurs would be expected every 2 years) for most of the rest of the country with the exception in northeast New South Wales (NSW) where it is closer to 1 event day per year. The standard deviation (per year) values for Elatent at each cell (Fig. 4b) suggest that the model has the greatest uncertainty around the northeast part of WA with standard deviations up to 3 event days per year.

The mean CAR term (Fig. 4c) is shown to be around zero for much of the country, with a slight tendency to small positive values in some regions. This suggests that the Li5-Shr1 index requires only minor adjustments to explain the observed event occurrence rate and, in some places, slightly underestimates the rate at which events occur. While not shown, there also appears to be higher standard deviation of CAR around the coasts compared to inland locations, which seems to be related to the mean shear values, which tend to be higher along the coast compared to neighbouring inland cells.

The spatial distribution of events changes during winter (Fig. 5a). The output from the LiMax-Shr3 model suggests there are little to no convective gust events occurring throughout most of Australia in the winter months. Similar counts are shown in the southern half of Australia with the exception of southwest WA and Tasmania (TAS), where there is closer to 1–2 event days per year occurring. The contrast in event counts between north and south Australia can be explained by the movement of the two predominate high-pressure systems in the Indian and Pacific Oceans equatorward. This results in strong subsidence in the north and a resultant lack of orographic lifting as the trade winds move the moist tropical maritime Pacific air mass parallel to the coast (Tapper and Hurry 1993). While in the south, the shifting of these high-pressure systems allows extratropical low-pressure systems and their associated cold fronts to move further north and interact more readily with the southern coastline. The peak in winter gust activity observed here follows closely those observed by Kuleshov et al. (2002) for general thunderstorm activity (based on lightning occurrence), with maxima in both Perth (southwest WA) and western TAS also observed in their analysis.

There is however high standard deviation in Elatent shown in Fig. 5b, with values up to 3 event days per year evident for several cells along the south coast of Australia. While not shown here, the mean values of Shr3 within these cells during winter show notably larger values than their surrounding cells. The CAR term (Fig. 5c) appears to be near zero for most of the country. The low CAR term is expected because event counts are so small, but marginally higher values are present in the southern part of the country albeit still small compared to other seasons.

In spring, the increase in gust event days noted in Sect. 2.1 across the county is clearly evident (Fig. 6a). This increase is caused by a shift in synoptic weather patterns as the high-pressure systems over the Indian and Pacific Oceans start to move south (Tapper and Hurry 1993) and begins to allow moist equatorial maritime air mass inland. The maximum number of convective gust events appear to occur in the interior of the continent, with numbers dropping off around the coast, particularly towards the south. Up to 4 event days per year can be seen in northeast NSW, with this local peak linked to the return of south-easterly trade winds bringing warm, moist Pacific and Tasman maritime air to this region during this time of year (Tapper and Hurry 1993). Moreover, orographic lifting of the Great Dividing Range along the east coast of Australia has the potential to aid in the maxima in event days seen in this region. There are also localized maxima in central Western Australia and the Northern Territory. These appear to occur around cells where AWS coverage is low compared to city centres but where multiple days with convective wind events have been recorded by those stations (Fig. 2c). Given the short duration of AWS records used, and the random but infrequent occurrence of strong convective gusts, such localized peaks are to be expected at some sites but are anticipated to be smoothed out as longer records become available. The standard deviation of Elatent (Fig. 6b) suggest that there is uncertainty with a large part of central Australia and the maximum in northeast NSW with standard deviation values approaching 3 event days per year.

The CAR term (Fig. 6c) seems to show mostly an underestimate of events across Australia (i.e. positive CAR), especially in the areas where event (Elatent) maxima are shown in Fig. 6a. There are however areas of overestimating along the coast in Queensland (QLD) and WA as well as over TAS.

The largest gust event counts are shown to occur in the summer months (Fig. 7a). Across most of the country there appears to be, on average, approximately 4 days per year with at least one convective wind event occurring in each cell. Event counts reaching up to 12 per year over northern WA are shown. This maximum extends east towards the Northern Territory (NT) border. Smaller maxima, around 8 days per year with at least one convective wind events appears in the NT and in the northeast corner of NSW, although these maxima don’t extend much beyond an individual grid cell.

The increase in events across the country during summer is largely attributed to the positioning of the intertropical convergence zone over the northern part of Australia and the associated convergence and uplift of very moist air, during this period. Frequency of these thunderstorms tends to decrease towards the south but exceptions have been noted over inland Western Australia where ‘dry’ thunderstorms are common (Kuleshov et al. 2002). In WA the general wind pattern is easterly bringing hot, dry continental air into the region. However, there is also the recurring of trade winds that bring warm, moist tropical maritime air from the Indian ocean into northern WA (Tapper and Hurry 1993). The interaction of these two different air masses has the potential to trigger severe convective weather, specifically the ‘dry’ thunderstorms, noted to occur in this region. As in spring, during summer the east coast of Australia experiences south-easterly trade winds that bring warm, moist Pacific and Tasman maritime air to this region and with it the potential for convection when aided by orographic lifting of the Great Dividing Range. This can be seen in the maxima of event days in the north east part of NSW in Figs. 6a and 7a.

Examining additional outputs from the model, the standard deviation of Elatent (Fig. 7b) is largest over northern WA, southern NT and even parts of western QLD. This is not surprising giving the magnitude of the mean Elatent values as well as the sparsity of observations in these regions (Fig. 1d). In contrast, the small maximum of Elatent over northeast NSW has a much smaller standard deviation for Elatent signifying less uncertainty in quantifying the process not explained by the explanatory variables. The mean CAR term in summer (Fig. 7c) shows the model underestimates the event day counts over the northeast part of WA and the southern part of the NT. There are however areas where the model overestimates event counts, those being the northern part of the NT and QLD, as well as over TAS and part of the southern coast of Australia. There is less deviation in the CAR term in cells that have AWS. While this may be expected, it is interesting how distinct the contrast is in summer.

**Fig. 8:**
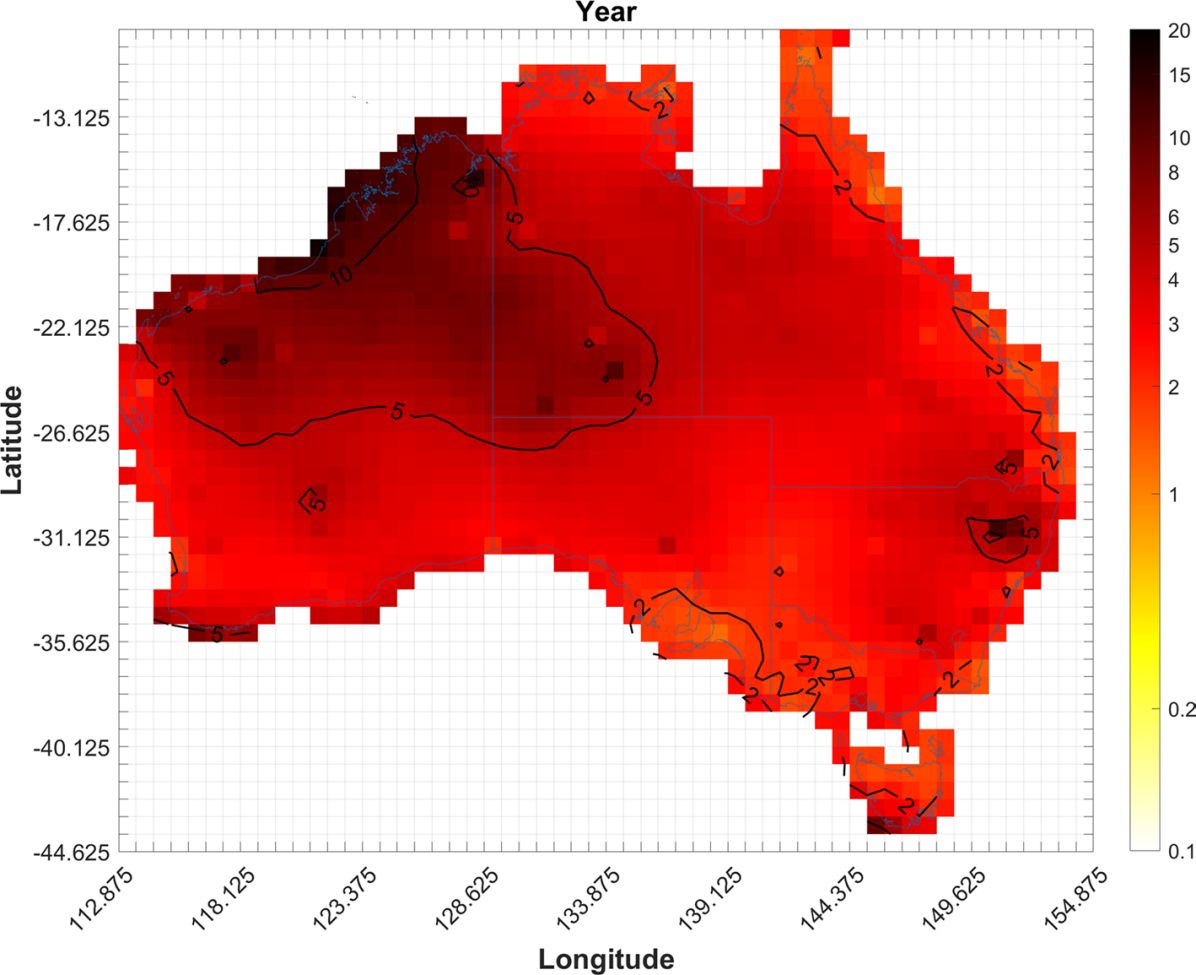
Average expected annual number of days with at least one convective gust per cell for the period 2005–2015

An aggregated count of the mean number of gust event days per year (i.e. summed across all seasons) is shown in Fig. 8. This figure shows that the majority of days with a severe convective wind gust appear to occur over the north half of WA, with about 15–20 event days per year, and extends to a lesser degree into the south half of the NT where around 10 event days are shown. The rest of the country sees between 1 and 5 event days per year, with the exception of a few cells over northeast NSW that have about 10–15 event days per year. Looking at the contribution of each season to the entire year (not shown) it is clear that the majority of events occur during the summer. With cells over northern WA, southern NT, and parts of QLD having up to 80% of their event days occurring in the summer. Much of the east coast of Australia, as well as southeast WA, SA, VIC, northern NT, and northern QLD, have about 40% of their event days occurring in the spring, whereas most of the country sees about 10–20% of their event days in autumn. Most cells have less than 10% of their event days occurring during winter with the exception of cells over the southern Australian coast and TAS where the percentage can reach up to 60%.

#### 1979–2015 reanalysis-based climatology

To accurately determine the hazard associated with any type of severe weather event, a long period of observations is generally necessary (Mason and Klotzbach 2013). While the results from the models discussed above provide a spatially complete climatology, it is only done so based on a short 11-year period. In this section, relationships developed in Sect. 2.3 and discussed in Sect. 3.1 are applied to 37-years (1979–2015) of ERA-Interim data, to investigate whether any biases or errors in the climatology may have been introduced due to the short period used up to this point.

**Fig. 9:**
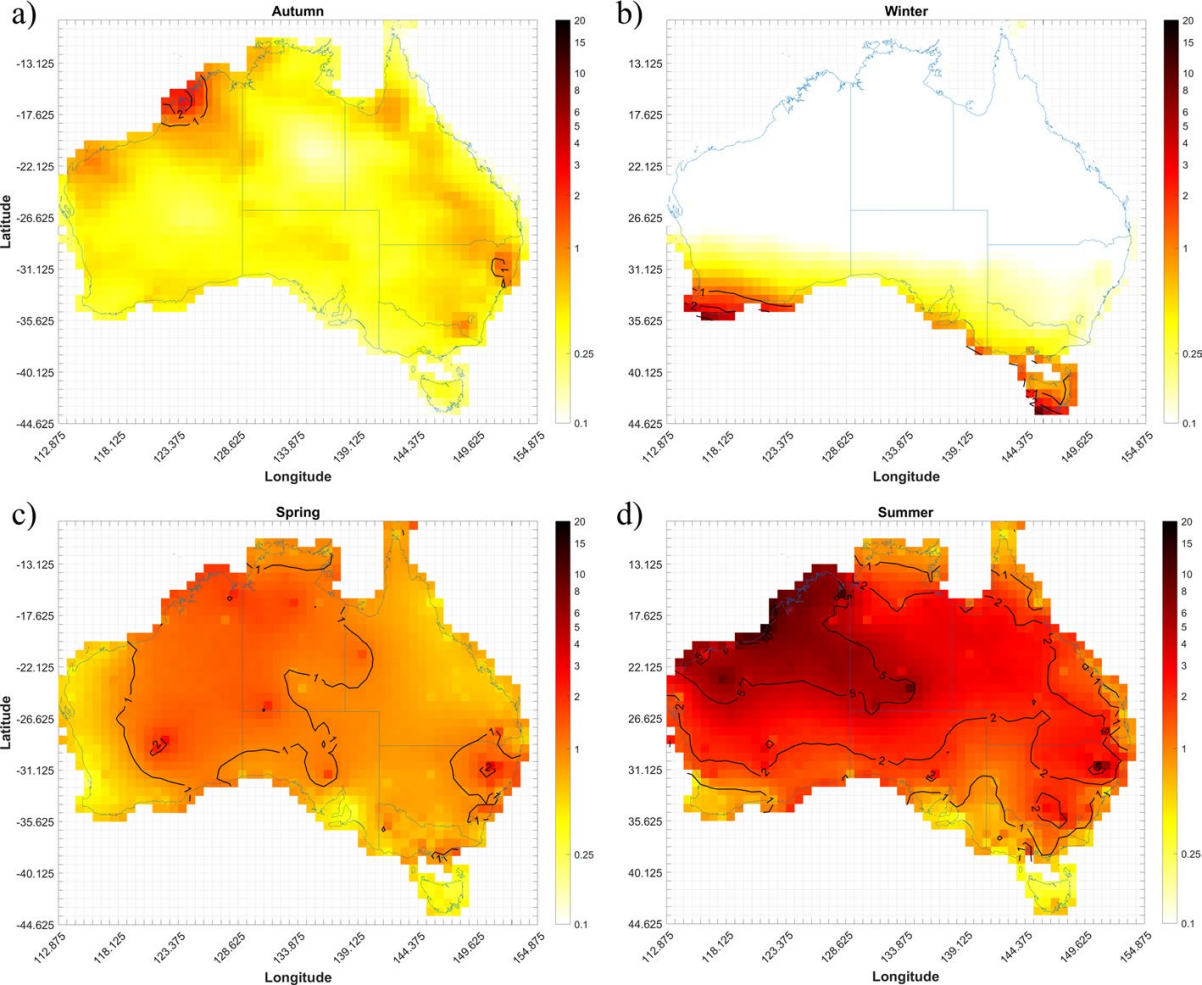
Average seasonal number of convective wind gust events for the period 1979–2015: **a** autumn (Li5-Shr1-Autumn model), **b** winter (LiMax-Shr3 -Winter model), **c** spring (Mburst-Spring model), and **d** summer (muCAPE-Shr1 -Summer model)

To extend the models to the longer ERA-Interim period (and without the AWS observations), Eq. 4 is solved for each season through a direct Monte-Carlo simulation using the distribution of each parameter ($\alpha_{0}$, $\alpha_{1}$, $\alpha_{2}$, $\alpha_{3}$, ${\text{CAR}}_{{\text{i}}}$) calculated by the hierarchical Bayesian models chosen in Sects. 3.1.1–3.1.4, and the mean seasonal index values calculated for the ERA-Interim reanalysis between 1979 and 2015. A random sampling of 50,000 years is used to give a distribution for the event occurrence rate, *λ*_*i*_, at each ERA-Interim grid cell *i*. Elatent is then solved in the Monte-Carlo simulation by taking the Poisson distribution of *λ*_*i*_. Results of this analysis are shown in Fig. 9 where average seasonal event occurrence rates (Elatent) are shown for autumn, winter, spring, and summer.

Comparing the climatology over the extended period of 1979–2015 (Fig. 9) with the period the Bayesian models were developed on (2005–2015) (Fig. 4–7), similar event counts and distributions of these counts seasonally, and across the country are evident. Over the entire year, there are slight variations to the event counts between the two periods, with an average absolute difference of ~ 0.03 events days per year, to a maximum difference of ~ 0.4 event days per year. Moreover, there is little visible difference to the spatial distribution of event occurrence. One of the more noticeable differences are, however, in winter, where the 1 event day per year line is shifted northward and a few cells approaching 2 event days per year can now be seen. The 1 event day per year line during spring is also found to extend more into SA for the 1979–2015 period compared to 2005–2015 and the 5 event per day line during summer expands more into NT for the 1979–2015 period.

When comparing these results with the climatologies based solely on weather station observations reported by Spassiani and Mason (2021), notably higher event counts are observed here; e.g. Spassiani and Mason (2021) observe 3 convective gust days per year in northwest WA, compared to up to 20 days found in this work. This difference is expected given their study provides a measure of event frequency at point locations (i.e. at an AWS station) while the current model provides a regional count measured over a 75 km × 75 km area, where many events will occur that won’t be recorded by all points within that region. The relative distribution of events across the country is similar though, but the incorporation of SWI through the Bayesian hierarchical model has provided more detail between the sparse network of weather stations thus providing greater information in those areas where stations don’t currently exist.

Comparisons with Brown and Dowdy (2021a) also show they find elevated gust event occurrence in the interior of Australia and a maxima along the east coast, similar to that shown in Fig. 8. However, Brown and Dowdy (2021a) do not show many events occurring over northern WA. Despite this, Kuleshov et al. (2002) and Dowdy and Kuleshov (2014) show peaks in lighting activity in this area, and Bedka et al. (2018) show a maximum in overshooting cloud-tops to occur in this area, all highlighting the potential for severe convective gusts to occur through the area despite the current lack of observations.

## Conclusion

This paper details the development of a spatially complete climatology of severe convective wind gusts across Australia. This is done while correcting for the biases found in typical report-based climatologies, where the density of observers (or observation sites in this research), or the period over which these observations are made can lead to an artificial maximum (often near population centers) or an artificial minimum in event counts (often in rural regions). Bayesian hierarchal modelling was utilized to correct for these biases while also using global reanalysis data (ERA-Interim) to interpolate between observations at weather station sites. Such a modelling approach builds upon similar successful climate studies in other parts of the world (e.g. tornadoes across North America in Cheng et al. 2016) but has been adapted here to study severe convective wind gusts in Australia.

Using weather station observations and 14 reanalysis-derived severe weather indices over the period 2005–2015, individual models were developed to explain the rate of severe convective wind gust occurrence for each of the four seasons (southern hemisphere autumn, winter, spring, summer) with results aggregated to estimate annual event occurrence rates. Results suggest that there are many more severe convective gust events occurring across Australia than are being observed. This is especially true in the interior of the continent, and in northern Western Australia. Broadly, a seasonal bias in the event day occurrence rate was observed, with a minimum number of events occurring during the autumn or winter months, a larger number of events occurring during the spring months and a maximum for all regions except the southern tip of Western Australia and Tasmania occurring in the summer. In addition, there is a shift in where convective gusts occur throughout the year with event days largely confined to the southern parts of Australia in the winter, and a shift to the north in the spring. There is also a small shift in the event maximum to the east during the summer, followed by a retreat south in autumn.

To overcome limitations that may exist in these derived climatologies due to the short observational record, each of the seasonal models were then run with severe weather indices calculated for the period 1979–2015 but in the absence of any AWS observations. This extended climatology showed similar event occurrence rate and spatial distributions to those shown directly from the Bayesian Hierarchical Modelling calculated for 2005–2015. Variability between models across the majority of Australia is within 2%, with no discernible spatial pattern visible.

While the use of a hierarchical Bayesian model with monthly mean SWI inputs was found to be useful, this approach – and the climatology developed here—does have scope for improvement. For example, use of an observational period greater than 11 years (with similar data quality), that captured more of the natural variability in event occurrence rate at a given location, is expected to lead to more reliable model convergence and may identify SWI with more explanatory power than those identified here. A longer observational period could also help limit the magnitude of the uncertainty in Elatent values, as evidenced through the large estimated standard deviation values, which could improve the model’s fits to the data and thus provide more certainty when interpreting the results from the Bayesian hierarchal models. In addition, only a limited number of seasonally-averaged severe weather indices were considered here. Use of SWI averaged over shorter periods (e.g. weekly or monthly), drawn from more resolute reanalysis, e.g. ERA5 (Hersbach et al. 2019) or the use of different indices, such as the logistical regression index recently develop by Brown and Dowdy (2021a, b), may also improve model performance as well as resulting climate estimates.

## Appendix 1 Variable descriptions

Table

**Table 11:** Short description for the variables used in the severe weather indices equations shown in Table 1

Variable	Description
$u$	*u*-component of the wind (east–west)
$v$	*v*-component of the wind (north–south)
$T$	Temperature in Kelvins
${\text{T}}_{{{\text{sfc }} \to {\text{i}}}}$	Temperature of the parcel lifted from the surface to the *i* pressure level
$g$	Gravitational constant
${\text{z}}_{{{\text{EL}}}}$	Height of the Equilibrium Level (EL)
${\text{z}}_{{{\text{LFC}}}}$	Height of the Level of Free Convection (LFC)
${\uptheta }_{{{\text{Ep}}}}$	Equivalent potential temperature of the parcel
${\uptheta }_{{{\text{Ee}}}}$	Equivalent potential temperature of the Environment
${\text{DLS}}$	Deep Layer Shear (0–6 km)
${\text{p}}_{{{\text{LFS}}}}$	Pressure of the Level aloft of Free Sink
${\text{p}}_{{{\text{sfc}}}}$	Surface pressure
$\alpha_{{\text{p}}}$	Specific volumes of the parcel
$\alpha_{{\text{e}}}$	Specific volumes of the environment
$\theta_{{\text{e}}}$	Equivalent potential temperature
${\text{sbCAPE}}$	Surface-based CAPE
${\text{ LI}}_{{{\text{sfc}}}}$	Surface-based Lifted Index
${ }\gamma_{{2{\text{m}} - 3{\text{km}}}}$	2 m-3 km lapse rate
${\text{VT}}$	Vertical Totals (850–500 mb temperature difference)
${\text{PWV}}$	Precipitable Water Vapour
${\text{TeD}}$	Theta-e Difference between the max and min values
$\overline{{{\text{V}}_{{1 - 3.5{\mathbf{km}}}} }}$	1–3.5 km mean wind

11 Present a list of the variables used in the equations of the severe weather indices shown in Table 1 (Sect. 2.2) along with a short description to define each symbol.
